# Mitogenome Announcement Characterization of the complete mitochondrial genome of golden tank goby, *Glossogobius aureus* (Perciformes: Gobiidae)

**DOI:** 10.1080/23802359.2020.1840943

**Published:** 2020-12-24

**Authors:** Muhammad Hilman Fu’adil Amin, Nazia Tabassum, Ah-Ran Kim, Dae-Sung Lee, Hyun-Woo Kim

**Affiliations:** a Industrial Convergence Bionix Engineering, Pukyong National University, Busan, Republic of Korea; b Department of Biology, Faculty of Science and Technology, Advance Tropical Biodiversity, Genomics, and Conservation Research Group, Universitas Airlangga, Surabaya, Indonesia; c Department of Marine Biology, Pukyong National University, Busan, Republic of Korea; d Department of Genetic Resources Research, National Marine Biodiversity Institute of Korea, Seocheon-gun, Republic of Korea

**Keywords:** Mitochondrial genome, *Glossogobius aureus*, Gobiidae, Indonesia

## Abstract

We applied next-generation sequencing (NGS) method to construct the complete mitochondrial genome of *Glossogobius aureus*. The obtained mitogenome of *G. aureus* (16,590 bp) exhibited a typical structure harboring 13 protein-coding genes (PCGs), 22 transfer RNAs (tRNAs), two ribosomal RNAs (rRNAs), and one control regions (D-loop). Most of mitochondrial genes are encoded on the heavy (H) strand, except for eight tRNAs and ND6. Unusual start codons were identified in COX1 (GTG) and ATP6 (TTG). Six genes (ND2, COX2, COX3, ND3, ND4, and CytB) were terminated by an incomplete stop codon (TA−/T–). A phylogenetic study showed that *Glossogobius* formed a clade distinct from other species in the subfamily Gobiinae. *G. aureus* was most closely related to *G. giuris* with 87.04% sequence identity among the four species in the genus *Glossogobius*.

As one of the richest members of species in Gobiidae, 29 species in the *Glossogobius* are currently accepted with more than 50 species in estimation (Hoese and Allen 2015). Despite the high species number, only three mitochondrial genome sequences in the genus have been reported in GenBank database (https://www.ncbi.nlm.nih.gov/genbank). We here report the mitochondrial genome from *Glossogobius aureus* collected from Indonesia using the next-generation sequencing (NGS) technique. G*. aureus* is widely distributed in the fresh and brackish waters of South Africa, Asia, and Oceania and this information would be useful to understand its genetic populations.

*G. aureus* was collected from the downstream of Porong River, East Java, Indonesia (7°33′07.6″S 112°50′44.9″E). The specimen was identified by the sequence identity (99.8%) to the database (MT981707) as well as the morphological characteristics in the previous study (Akihito and Meguro 1975). The specimen and its genomic DNA were stored at both Department of Biology, Universitas Airlangga, Indonesia, and the Marine Biodiversity Institute of Korea (MABIK GR00004011). The mitochondrial DNA (mtDNA) was isolated from the skeletal muscle using the mitochondrial DNA isolation kit (Abcam, Cambridge, UK). After sheared by Covaris M220 Focused-Ultrasonicator (Covaris Inc., San Diego, CA), the DNA was used for the library construction using TruSeq^®^ RNA library kit V2 (Illumina, San Diego, CA), which was further sequenced by MiSeq (Illumina, San Diego, CA). The mitogenome sequence of *G. giuris* (NC_036674) was used as a reference for assembling the raw reads using Geneious software (Kearse et al. 2012). Secondary structures of tRNAs were predicted using tRNAScan-SE online software (Lowe and Chan 2016). A phylogenetic tree of *G. aureus* and other goby fishes was constructed using MEGA X Software with minimum evolution (ME) algorithm (Kumar et al. 2018).

The circular mitochondrial genome of *G. aureus* (MT968499) was 16,590 bp in length. It showed a general arrangement of mitochondrial genome in the vertebrates, including 13 protein-coding genes (PCGs), 22 transfer RNAs (tRNAs), two ribosomal RNAs (rRNAs), and a control region (D-loop). Twenty-eight genes were located on the heavy (H) strand, while nine genes were on the light (L) strand. Besides COX1 (GTG) and ATP6 (TTG), all the other PCGs were initiated by ATG. Incomplete stop codons (T–– or TA−) were identified in six PCGs, including ND2, COX2, COX3, ND3, ND4, and CytB. The length of 22 tRNAs varies from 66 to 76 bp and all of them formed typical clover structures except for tRNA^Ser-GCT^, which lacked its D-arm. A phylogenetic analysis using 17 mitogenomes in the Gobiidae showed that four species in the *Glossogobius* formed a clade distinct from other species in the subfamily Gobiinae (Figure 1). Among the four *Glossogobius* species, *G. aureus* was most closely related to *G. giuris* with 87.04% identity, followed by *Glossogobius circumspectus* (80. 52%). The mitogenome sequence of *G. aureus* would provide a useful information for its scientific conservation.

**Figure 1.**
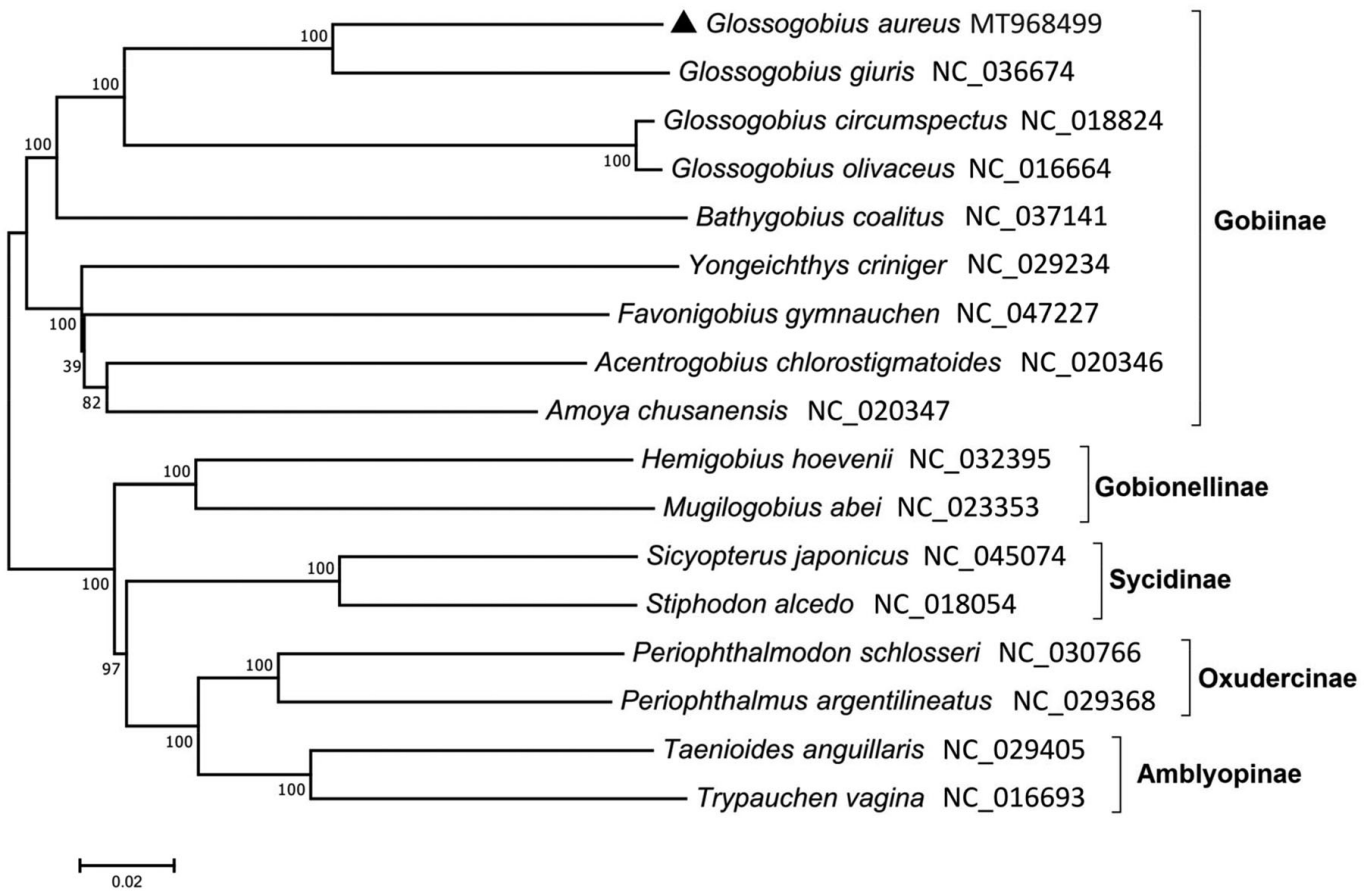
A phylogenetic tree of gobiid fish species constructed with the mitogenomes using ME algorithm. A number at each node indicates bootstrap value (1000 replicates).

## Data Availability

The data that support the findings of this study are available in GenBank database at *Glossogobius aureus* (GenBank Number: MT968499) https://www.ncbi.nlm.nih.gov/nuccore/ MT968499.1.
